# Excision of the Knee and Amputation of the Thigh for Disease of the Knee-Joint

**Published:** 1876-08

**Authors:** 


### Excision of the Knee and Amputation of the Thigh for Disease of the Knee-Joint.

At the last meeting of the Philadelphia College of Physicians, Dr. John Ashhurst read a most interesting and valuable paper on the above subject. The paper began with the histories of ten cases of excision and five of amputation, all performed by Dr. Ashhurst himself. Excision was performed three times for arthritis with partial anchylosis, twice for arthritis with abscess, once for arthritis following small-pox, and four times for gelatinous arthritis. Nine of these cases have recovered with useful limbs, one however still wearing a splint, as the operation was but recently performed, and the tenth case is convalescent and promises to have as favorable a result as the other nine. Amputation of the thigh was performed in five cases that did not admit of excision on account of the extent of the disease or of visceral complications. One of these cases only proved fatal. This patient was fifty-one years of age, and a man of intemperate habits; and the operation was performed for acute destructive inflammation of the knee-joint following an injury to the leg. In all the other fourteen cases the arthritis was chronic, and the patients were between five and eighteen years of age.

After concluding the account of this remarkable series of cases, Dr. Ashhurst put his remarks in the form of answers to the following questions:

I. When should the surgeon abandon expectant measures in the treatment of knee-joint disease, and what may be considered the indications for a resort to operation?

II. Operative interference having been resolved upon, how shall the surgeon decide between excision and amputation?

III. When an attempt is made to save the limb by excision, how shall the operation be performed, and what shall be the after-treatment?

I. In answer to the first question it must be premised that most cases of inflammation, even of the larger joints, dependent on blows, sprains, or internal causes, are amendable in the early stages to treatment by rest and extension. Unfortunately, however, the case often appears at first so trivial that it is neglected until it is too late for the above treatment to be efficacious. Of the ten cases of excision reported above, in only one had the disease lasted less than one year, the duration ranging in the other cases from one to six years. Dr. Ashhurst does not deny that some of these cases might ultimately have got well without an operation, but the limbs secured by the operation were immeasurably better and more useful than the withered and deformed limbs resulting from the so-called spontaneous cures. In deciding on the propriety of an operation in any particular case, the age and general condition of the patient, and the duration and stage of the affection, must be considered. Excision of the knee is not very successful in children under five years of age; these suffer more from the confinement than older children, and, Dr. Ashhurst thinks, are also more liable to the insidious development of tuberculous and other constitutional diseases. In persons past the prime of life excision of the knee is so fatal, that it should be entirely discarded, and amputation resorted to where an operation is absolutely indicated. The most favorable age for excision is from five years to puberty.

The presence of visceral disease, as a rule, positively contra-indicates excision. An operation is seldom required in recent cases, in which rest, extension, and proper constitutional and local treatment will usually effect a cure. Dr. Ashhurst maintains that these chronic joint affections, though unquestionably local diseases, are not of exclusively local origin, and that they require constitutional as well as local treatment. Finally, no operation should be performed as long as the disease is confined to the synorial membrane, or, even when all the tissues of the joint are involved, as long as the integrity of the part is maintained and a hope of preserving a useful joint remains. "But when the relaxed condition of the joint and the occurrence of consecutive dislocation show that the crucial ligaments and semilunar cartilages have disappeared; when the limb is contracted and helpless, and the patient give a history of repeated relapses from comparatively slight injuries; or, on the other hand, when the doughy semi-elastic character of the swelling shows the existence of gelatinous arthritis (the typical 'white swelling' of the old writers), an operation may be properly resorted to, even if the limb be at the time in a quiet condition. When in addition the joint is in a state of suppuration, and still more if there be caries of the articular surfaces, an operation may be considered (other things being favorable) as almost imperative." The operation may be performed comparatively early in cases of gelatinous arthritis in which there is hardly any tendency to spontaneous recovery.

II. Amputation should be performed in preference to excision when the patient is too young or too old, when there are visceral complications, when the disease is to too extensive to allow of its entire removal without taking away so much bone as would materially impair the usefulness of the limb, and when the question of the time required for recovery is important.

III. In performing the operation of excision, Dr. Ashhurst prefers the transverse incision across the front of the joint, just below the patella. He dissects back the skin and superficial fascia to the level at which he means to use the saw, then cuts directly down to the bone, and removes condyles, patella, and the diseased tissues together. The bones are divided from below upwards by means of a Butcher's saw with blade reversed. The femur, in order to retain the natural inclination of the limb, should be sawn in a plane which as regards the axis of the femur is oblique from behind forwards, and from within outwards. The tibia should be sawn in a plane transverse to its long axis, with a slight anterior obliquity. The epiphyseal junctions should not be interfered with in children; it is sufficient to remove a slice half an inch to an inch thick from the tibia. Any shreds of disorganized tissue may be cut away, but the floor of the wound must be left intact. The patella should always be removed. Tenotomy of the ham-string muscles may be necessary before the limb can be brought into position. Before the patient emerges from the anæsthesia, a posterior splint, bracketed opposite the knee, to allow of daily dressing of the wound, should be applied, and left undisturbed for at least a fortnight, and better still for six weeks, at the end of which time the process of bony union will be well advanced. A straight position is the best for the limb, on account of the shortening produced by the operation.—*Medical Record.*

:o:

Elephantiasis of the Nose.—Dr. N. Matteucci reports (*Bull. delle Scienze Mediche*) an interesting case of a voluminous elephantiasis of the nose successfully removed by Prof. Rizzoli. Very few cases of this deformity have thus far been observed. Three cases have been reported by Billroth, one by Gurlt, one by Barilli, and three by Vangel. The individual operated on by Rizolli began to suffer from this disease at the age of forty-three. It commenced with the production of small tumors consecutive to an erysipelas of the face. He objected at first to an operation, and it was only when the deformity had progressed so far as to render him a laughing-stock to everybody, that he consented to its removal. There were a number of tumors, three of which were quite large, the total weight was 280 grammes. The diagnosis was soft fibroma of the derma and subcutaneous connective tissue in a condition of proliferation, with hypertrophy of the sebaceous glands. A perfect cure was obtained.—*Lo Sperimentale*, April, 1876.—*Medical Record.*

